# Trial-Based Cost-Utility Analysis of Icotinib versus Gefitinib as Second-Line Therapy for Advanced Non-Small Cell Lung Cancer in China

**DOI:** 10.1371/journal.pone.0151846

**Published:** 2016-03-25

**Authors:** Chunxiang Zhang, Hongmei Zhang, Jinning Shi, Dong Wang, Xiuwei Zhang, Jian Yang, Qizhi Zhai, Aixia Ma

**Affiliations:** 1 School of International Pharmaceutical Business, China Pharmaceutical University, Nanjing, Jiangsu Province, China; 2 Department of Pharmacy, Nanjing Jiangning Hospital, Nanjing, Jiangsu Province, China; 3 Department of Blood Transfusion, Nanjing Jiangning Hospital, Nanjing, Jiangsu Province, China; 4 Department of Respiratory, Nanjing Jiangning Hospital, Nanjing, Jiangsu Province, China; Catalan Institute of Oncology, SPAIN

## Abstract

**Background:**

Our objective is to compare the cost-utility of icotinib and gefitinib for the second-line treatment of advanced non-small cell lung cancer (NSCLC) from the perspective of the Chinese healthcare system.

**Methods:**

Model technology was applied to assess the data of randomized clinical trials and the direct medical costs from the perspective of the Chinese healthcare system. Five-year quality-adjusted life years (QALYs) and incremental cost-utility ratios (ICURs) were calculated. One-way and probabilistic sensitivity analyses (PSA) were performed.

**Results:**

Our model suggested that the median progression-free survival (PFS) was 4.2 months in the icotinib group and 3.5 months in the gefitinib group while they were 4.6 months and 3.4 months, respectively, in the trials. The 5-year QALYs was 0.279 in the icotinib group and 0.269 in the gefitinib group, and the according medical costs were $10662.82 and $13127.57. The ICUR/QALY of icotinib versus gefitinib presented negative in this study. The most sensitive parameter to the ICUR was utility of PFS, ranging from $-1,259,991.25 to $-182,296.61; accordingly the icotinib treatment consistently represented a dominant cost-utility strategy.

**Conclusions:**

The icotinib strategy, as a second-line therapy for advanced NSCLC patients in China, is the preferred strategy relative to gefitinib because of the dominant cost-utility. In addition, icotinib shows a good curative effect and safety, resulting in a strong demand for the Chinese market.

## Introduction

Recently, the epidermal growth factor receptor (EGFR) expressed in the solid tumor of epithelial origin, has been demonstrated as one of the most important oncogenic drivers. Overexpression of EGFR has been reported and implicated in the pathogenesis of many human malignancies, including NSCLC[1]. Treatment with EGFR-tyrosine kinase inhibitors(TKIs) has shown significant survival benefit for advanced NSCLC patients with EGFR(+). Advanced NSCLC with mutated epidermal growth factor receptor have significant responses to EGFR TKIs, it appears to be a significant survival advantage [2,3]. It is reported that patients with EGFR(+) is sensitive to EGFR TKIs and EGFR(+)occurs more frequently in Asian patients than white patients[4,5,6,7].

The oral anti-cancer drugs that inhibit EGFR, gefitinib (Iressa) was the first EGFR TKIs used for solid tumor therapy and it had outstanding performance in the clinical treatment of the past[8,9]. The icotinib (Commana, Betta Pharmaceuticals Co. Ltd, Hangzhou, China) was approved by the Chinese State Food and Drug Administration (SFDA) in June 2011. Previous studies had shown that icotinib had a good efficacy and tolerability as monotherapy for EGFR(+) NSCLC[10,11,12,13]. Before the advent of icotinib, only gefitinib and erlotinib were available in the Chinese market while both the majority of patients and medical insurance institutions could not afford them due to the highly acquisitive prices, so there was a need in China to develop innovation drugs including icotinib with independent intellectual property rights. In this study, we evaluated the cost-utility of icotinib and gefitinib as a second-line treatment for advanced NSCLC by pharmacoeconomics methods.

## Materials and Methods

Data and medical history were from clinical trials [12] that were implemented in 27 centers in China. We developed the Markov model to reflect the progression of advanced NSCLC.

### Model Structure

We used a Markov decision model to assess the 5-year clinical outcomes and economic investments of two targeted drugs. 5-year time horizon can reflect almost all the disease progression and the survival benefits from second-line treatment for the advanced NSCLC patients[12,14].

**Fig 1:**
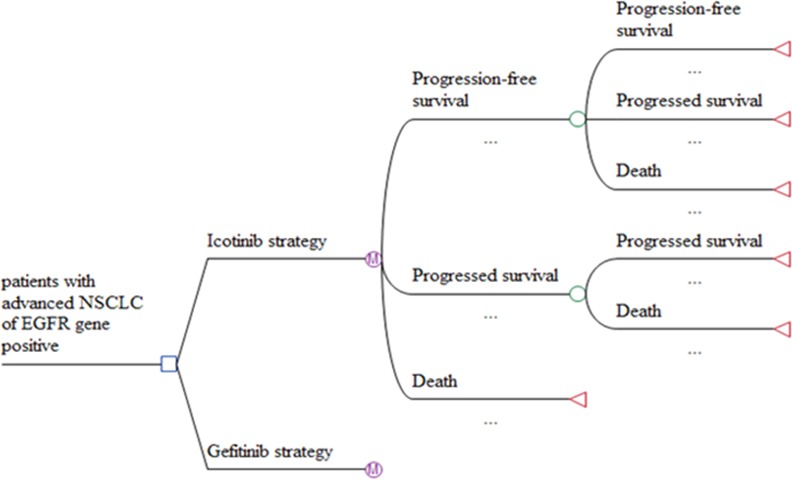
Markov decision tree model structure of icotinib and gefitinib strategies for the treatment of advanced NSCLC.

The decision model for NSCLC consisted of three mutually exclusive health states: progression-free survival (PFS), disease progression (DP) and death (Fig 1). At the starting point, all the patients were in the PFS state and the length of each cycle was 21 days for the two groups. As time followed, patients transferred gradually from the PFS→DP, DP→death. Once patients entered a new state, they couldn’t return the original state. At the end of each cycle, patients might remain in the PFS, or progress into DP or death and the death state was the final one. The entire patients entered the death state as time went on long enough.

We built a Markov model to estimate the direct clinical costs, and quality-adjusted life years (QALYs) gained from the practical trials[12]. The results were presented as incremental cost-utility ratios (ICURs).

A hypothetical cohort that was clinically similar to the advanced NSCLC patients in the non-inferiority trial entered in the model[12]. The hypothetical patients, at age 28–75, histologically or cytologically confirmed Stage IIIB or IV NSCLC with progression after at least one platinum-based chemotherapy regimen were randomly assigned to receive icotinib(n = 199) or gefitinib (n = 196).

Medication method is as follows:

1. Icotinib treatment, 125 mg/time, 3 times per day, until disease progression or unacceptable adverse reactions occur;
2. Gefitinib treatment, 250 mg per day, until disease progression or unacceptable adverse reactions occur.

It was assumed that all the patients got active treatment with docetaxel 75mg/m^2^ on day 1 of each 3-week cycle for 6 cycles after the disease progression[15,16].

### Collection and Analysis of Clinical Data

The clinical data were extracted from the randomized, double-blind phase 3 non-inferiority trial[12].A Weibull model was fitted to the data extracted from the Kaplan-Meier survival PFS and OS curves to estimate transition probabilities for each 21 days. The validity can be determined by comparing PFS and OS calculated from the model with median PFS and OS obtained from actual trial.

Two-parametric Weibull survival analyses using STATA package version 12.0 (Stata Corp, College Station, Texas, USA) were fitted to the PFS and OS data from the trial, then we got the two key parameters of scale (λ) and shape (γ). The scale parameter (λ) is related to the measurement unit of time. The shape parameter (γ) defines the hazard function to increase or decrease with increasing time; γ>1 indicates an increase of the hazard rate in nonlinear pattern. The time-dependency transition probability among PFS, DP and death could be gotten by the following formula:

$$P\left(t\right)=1-\exp\left\{\lambda {\left(t-1\right)}^{\gamma }-\lambda t^{\gamma }\right\}$$

Because our economic analysis was based on model technology and literature review, this study did not require the ethical approval from the Medical Ethics Committee of Nanjing Jiangning Hospital.

### Medical Costs

Medical costs data were from the perspective of Chinese health care system. Only direct medical costs were considered, while indirect costs of productivity lost, mental illness, or transportation etc were excluded. In this study medical costs mainly included: drug, treatment for adverse drug reactions (ADR), and follow-up examination in DP state. The prices of icotinib and gefitinib were $449.80/unit (125mg*21pills) and $865.94/unit (250mg*10 pills) respectively. In every 21-day cycle, 63 pills of icotinib or 21 pills of gefitinib were supplied to each group. Hence, the costs were $1,349.40 or $1,818.48 accordingly (The prescription was mentioned above). The high price of each drugs was a key challenge for most advanced NSCLC patients. Both manufacturers had promised that all qualified patients could get free drugs without any charge. AstraZeneca announced the gefitinib Patients Assistance Program (GPAP) to provide help to eligible patients. Patients who would be eligible to receive treatment must meet the requirements: 1) patients had been taking gefitinib continuously for 5 months and disease did not progress; 2) they were disabled patients. Beta Pharma offered free icotinib for all NSCLC patients who had taken icotinib continuously for 6 months without tumor progression.

Because ICUR was mainly caused by the difference of clinical outcomes and costs, it was proper to exclude any item that had no difference between the two groups such as physician visits, care costs, biochemical test costs, computed tomography (CT) examination costs. The main costs per cycle mainly come from prescribed medication and treatment of ADR. The occurrence of ADRs in target therapy was less than that of the chemotherapy treatment. In our clinical trial, The ADRs utility was referenced from literature review[15,17]. It is well known to oncology experts that severe pain and hemoptysis listed in experimental articles were caused by cancer itself instead of ADR, therefore the cost of treating this symptom was not considered.

### Utility Values

In this study, health state utility values were obtained from the literature [18,19].We assumed that health utilities of patients in China were equivalent to that of the UK. Because of less ADRs caused in oral targeted therapies, health utilities value were higher than those of chemotherapy. The health utilities were calculated based on previous work. The utility value of PFS in targeted drugs therapy was 0.673, and the corresponding utility of PS was 0.473. If the same ADRs occurred in both groups, we assumed the reduced utility value would be similar. The most common ADRs in this trial were listed as diarrhea, nausea, rash, and raised aminotransferase, and the corresponding utility value were 0.606, 0.605, 0.621 and 0.639.

### Calculation

The cost data, the incidence of ADR, and the model parameters calculated from utility of various states were summarized in Table 1. We input the model parameters into TreeAge software and performed the one-way sensitivity analysis and the probabilistic sensitivity analysis (PSA) at the same time.

**Table 1:** Baseline costs, risks and utility values of icotinib and gefitinib in advanced NSCLC patients in China. Data are in 2014 US dollars.

Parameter	Base case	Range	Source
Drug costs ($ 21 days/one unit)			
Icotinib	1,349.40	1349.40	Market
Gefitinib	1,818.48	1610.90	Lognormal
Routine follow-up of patients per unit[15]	57.96	50.64	Lognormal
Docetaxel, 1 ampule at 20mg[15]	108.72	74.96	Local
Costs of ADRs ($)			
Diarrhea	5.18	4.14/6.22	±20%
Nausea	13.61	10.89/16.33	±20%
Rash	5.50	4.4/6.6	±20%
Raised aminotransferase	216.35	173.08/259.62	±20%
Probability of serious ADR events(%)			[12]
Diarrhoea	2	1.4	±30%
Rash	1	0.7	±30%
Raised	0.5	0.35	±30%
Raised	1	0.7	±30%
Rash	0.5	0.35	±30%
Nausea	0.5	0.35	±30%
Utility values			
PFS on oral therapy[20]	0.673	0.27/0.80	
DP [20]	0.473	0.19/0.56	
Diarrhoea in gefitinib group [20]	0.606	0.24/0.73	
Rash in gefitinib group[20]	0.621	0.25/0.74	
Rash in icotinib group [20]	0.621	0.25/0.74	
Nausea in icotinib group[20]	0.605	0.543/0.571	
Discount rate(%)[21]	3	0/8	Fixed in PSA

Abbreviation: NSCLC, non-small cell lung cancer; ADR: adverse drug reaction; PFS, progression free survival; DP, disease progression, PSA = Probabilistic sensitivity analysis.

### Ethics Statement

Our study used the method of mathematical model, not an actual clinical trials, and our study didn’t violate ethics restrictions.

## Results

### Model Validation

Survival analyses from the Markov model and the clinical trial were presented in Table 2. The clinical trial indicated that the median PFS and OS were 3.4 and 13.9 months in gefitinib group, compared with 4.6 and 13.3 months in icotinib group respectively. In the Markov model, the corresponding data of gefitinib group were 3.5 and 14.0 months and the data of icotinib were 4.2 and 13.6 months. The differences of PFS and OS data between the model and the clinical experiment were slight, indicating the reliability and accuracy of this mathematical model.

**Table 2:** The median survival time of icotinib and gefitinib as second-line therapy for advanced NSCLC patients. (month).

Treatment	Trial	Model	Difference
**Progression-freeSurvival**			
Icotinib	4.6	4.2	0.4
Gefitinib	3.4	3.5	-0.1
**Overall survival**			
Icotinib	13.3	13.6	-0.3
Gefitinib	13.9	14.0	-0.1

### Parameters of Weibull Models

We used STATA package (version 12) to fit the data of PFS and OS from the trial, and then we got two key parameters of scale (λ) and shape (γ). We wrote a survival probability formula and calculated the survival of different periods. Weibull parameters are as Table 3 follows:

**Table 3:** Parameters of Weibull models fitted to the Kaplan-Merier survival curve in the trial.

Treatment	Scale,Mean(SE)	Shape,Mean(SE)	Adjusted R^2^*	Correlation Coefficient
**Progression-free survival**				
Icotinib group	0.9359(0.0528)	-5.0757(0.3160)	0.9921	0.9960
Gefitinib group	0.94(0.0518)	-5.2677(0.3375)	0.9845	0.9925
**Overall survival**				
Icotinib group	1.1664(0.0770)	-7.3062(0.4963)	0.9963	0.9982
Gefitinib group	1.1938(0.0811)	-7.5374(0.5235)	0.9965	0.9982

* Estimated for the extracted data with model base projection.

### The Medical Investment and Health Outcomes of 5 Years

We imported the model parameters into the TreeAge software with the baseline of 5 years, and assessed the 5-year clinical outcomes and economic investments of two targeted drugs. The 5-year QALYs was 0.279 in the icotinib group and 0.269 in the gefitinib group, and the accordingly medical costs was $10662.82 and $13127.57. The ICUR/QALY of icotinib versus gefitinib presented negative in this study. According to the WHO's recommendations[20], it was significantly economic when ICUR/QALY was less than one time of Gross Domestic Product(GDP), however the ICUR/QALY was negative in this study. We could say that icotinib had absolute advantage in second-line treatment of advanced NSCLC compared with gefitinib.

### Sensitivity Analyses

We used one-way sensitivity analysis and PSA to assess the effect of model parameters on the results and the influence degree on the results.

**Fig 2:**
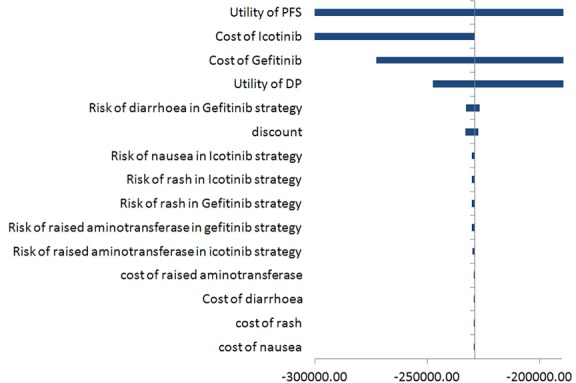
Incremental cost-effective ratio (ICUR) tornado diagram.

As shown in Fig 2 of one-way sensitivity analysis, we obtained the sensitive parameters including utility of PFS, cost of icotinib per cycle (≤ 9), cost of gefitinib per cycle (≤7), utility of DP (0.19–0.56), risk of diarrhea in icotinib group (1.4–2.6), and discount (0–8%) in a descending order. Among them, utility of PFS was the most sensitive parameter that affected the ICER result, ranging from $-1,259,991.25 to $-182,296.61.

**Fig 3:**
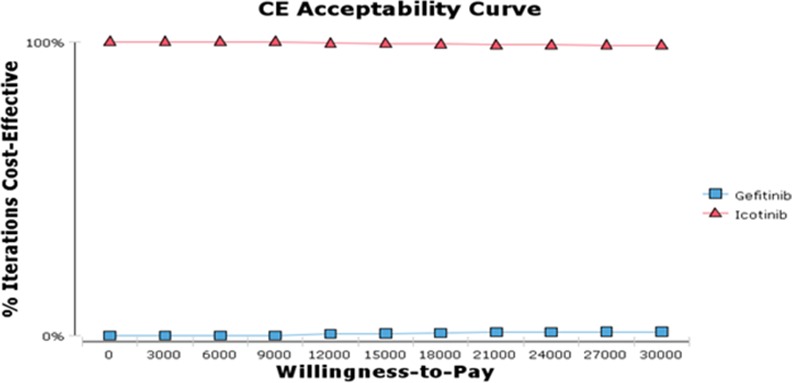
Cost-utility Acceptability Curve of Icotinib and Gefitinib.

We also conducted PSA to examine cost-utility acceptable curve under different Willingness-To Pay (WTP) (Fig 3). In general, the probability about different treatment strategies having cost- utility advantage will not vary with an increasing WTP. Fig 3 indicated the situation of acceptable curve about cost and utility of the second-line treatment of advanced NSCLC at different WTP. In spite of an increasing WTP, icotinib approach was consistently a preferred strategy. According to the WHO's recommendations, it was significantly economical when the ICUR/QALY ratio was less than one time of Gross Domestic Product (GDP)[20]. As a matter of fact, the ICUR/QALY ratio was negative in our study. There was no doubt that icotinib had a competitive advantage in second-line treatment for advanced NSCLC compared to gefitinib.

## Discussion

In developed or developing countries, lung cancer is the leading cause of cancer-related death in recent years. Statistics indicated that there were 219,440 newly diagnosed cases and 159,390 deaths in the US in 2009. NSCLC is the most common, accounting for approximately 80% form of lung cancer cases[2]. Chemotherapy has been the cornerstone of treatment for NSCLC for long periods of time. Targeted therapy is an effective treatment and can effectively prevent further deterioration. The development of EGFR TKIs for clinical use and the newly discovered EGFR mutations in NSCLC have resulted in a considerable number of researches and publications in this field. To date, EGFR mutation analysis by direct genomic sequencing has been the most studied and the reliable method to predict response to EGFR TKIs. EGFR TKIs might be particularly beneficial in Chinese because EGFR mutations are more common in Asian population (40–50%) than in White people (10%)[21,22,23]

Gefitinib was the first targeted therapy drug, providing a treatment approach for patients with advanced NSCLC in China. Though targeted drugs is particularly significant in the fight against cancer, but high price makes gefitinib unaffordable for the vast majority of the Chinese patients. Prior to the advent of icotinib, only gefitinib and erlotinib were available in the Chinese market while both the majority of patients and medical insurance institutions could not afford them due to the highly acquisitive prices. Fortunately, with the appeal of the China Charity Federation, many targeted drugs manufacturers launched free donations to the eligible patients. Zhu et al[24] compared the difference of the economic burden of patients participating in the GPAP program and non-participants maintaining the anti-cancer therapy after first-line treatment. For example of one-year treatment, the drug cost of gefitinib group participating in the GPAP program was only $8,980.4. Without GPAP, the cost would be $13,775.3 with a 53% increase. The long time treatment might make a more dramatic difference in drug cost, suggesting the critical importance of free donations to the patients.

Icotinib is a very few innovative drugs with its independent intellectual property rights of China. China has a particularly high demand for anti-lung cancer drug because of the high prevalence of lung cancer. Icotinib, an orally administered EGFR TKI, has potent antitumour activity in vitro and in vivo[25]. Moreover, icotinib showed high specificity and selectivity to its target EGFR in a preclinical kinase profiling study: only EGFR mutants were inhibited among 88 kinases profiled. The clinical benefit of icotinib was shown in a phase 2 study in which 103 patients were enrolled at ten dose levels. Objective responses were noted in 29.2% of patients, disease control were achieved in 78.1%, three complete responses were reported.[11,26,27]

As of now, this is the first evaluation of the cost-utility of icotinib versus gefitinib as second-line therapy for advanced NSCLC based on clinical trials using the Markov model. Although there are no differences in efficacy outcomes between icotinib and gefitinib, icotinib must be administered 3 times a day with a foreseeable lower patient compliance compared to once daily schedule of gefitinib. Patients in icotinib group owned high EGFR WT ratio(57%)[12] and this part of patients would progressed quickly. It makes icotinib group pay for a shorter time in the previous six months than that of gefitinib group in the previous 5 months. This should be a reason that icotinib group costed less.

There are many inevitable limitations in our study. First of all, we could not collect a rather huge amount of treatment-specific cost data for our research. Clinical physicians and drug manufacturers mostly focused on the clinical heath outcomes, and they seldom paid more attention to the medical cost data. Second, this study might authentically reflect the real situation if using the authoritative Chinese health utility, not the utility data obtained from Nafees’ research carried out in British. Thirdly, it was practically impossible to carry out the long-term clinical trials. To achieve the long-term health outcomes, researchers usually designed model methods to extrapolate the survival data. Though model offers a tool to analyze and predict the natural development of the disease, while it would not accurately reflect the patient's entire disease progression in the actual clinical trials. Last but not least, during the period of actually clinical cancer treatment, many adjuvant therapies including immunotherapy, traditional Chinese medicine and so on could have significant influences on the cost of cancer therapy. Particularly, traditional Chinese medicine was widely accepted in China, and it was commonly applied in clinical practice including the treatment of advanced NSCLC. To simplify our evaluation, we excluded those adjuvant therapies.

Since the marketing of icotinib, the situation that the Chinese market was monopolized by imported drugs has changed. As a result, the prices of targeted drugs decreased. This helps meet the increasing demand of targeted drugs for the treatment of advanced NSCLC in the Chinese market. This fact highlighted the importance that China should independently develop innovation drugs of their own to ensure affordable prescription drug coverage.

In developed or developing countries, medical resources are comparatively limited, but valuable to safeguard the health. Health policy makers need to allocate resources in the most cost-utility manner, yielding the best health outcomes for the limited resources. We first launched the economic evaluation of icotinib for second-line therapy for advanced NSCLC using a mathematical model based on the actual clinical trials. Our findings will provide information that is beneficial to the rational drug use, the selection of a particular drug in patients, health policy decision makers from the government.

**Conclusions:** As a second-line therapy for the advanced NSCLC patients in China, icotinib shows better cost-utility comparing with gefitinib.

## Supporting Information

S1 File Data processing. (ZIP)
